# Incomplete follow-up of positive HPV tests: overview of randomised controlled trials on primary cervical screening

**DOI:** 10.1038/sj.bjc.6605771

**Published:** 2010-07-13

**Authors:** M Rebolj, E Lynge

**Affiliations:** 1 Department of Public Health, University of Copenhagen, Øster Farimagsgade 5, DK-1014 København K, Denmark

**Keywords:** cervical cancer, screening, human papillomavirus, cytology, follow-up compliance, randomised controlled trial

## Abstract

**Background:**

It has been suggested that adjustment for incomplete compliance with follow-up in women with positive human papillomavirus (HPV) tests would be appropriate for estimating the true sensitivity of cervical screening with HPV testing. We assessed the compliance and its impact on ⩾CIN3 detection in all eight randomised controlled trials (RCT) with published baseline-round data.

**Methods:**

We extracted data on recommended follow-up procedures, follow-up compliance, and ⩾CIN3 detection for both arms of each RCT, and assessed their correlation.

**Results:**

Compliance with a direct referral for colposcopy was around 90% in all RCTs, whereas compliance with repeated testing among HPV-positive/cytology-negative women was around 60% in three RCTs and 73% in one RCT. Detection of ⩾CIN3 was significantly increased in two out of six RCTs with reported data. The correlation between compliance with follow-up in HPV-positive women and relative ⩾CIN3 detection was 0.48 (*P*=0.33).

**Conclusion:**

There is at present scant evidence to support the view that the measured sensitivity of HPV screening is a simple reflection of compliance with follow-up. Adjustment of measured cervical intraepithelial neoplasia detection on the basis of compliance data may not always be justifiable, and if adjustment is made, it should be used very judiciously.

Organised screening programmes using cytological testing are proven to reduce both the incidence of and the mortality from cervical cancer. However, screening with this method is needed every 3–5 years, and even then the disease is not completely eradicated in screened women (IARC, 2005). Human papillomavirus (HPV) DNA screening seems to be an attractive alternative to cytology screening. This was first supported by split sample studies in which more high-grade cervical intraepithelial neoplasia (CIN) was detected with HPV DNA screening than with cytology screening (Cuzick *et al*, 2008). Similar findings came from some of the randomised controlled trials (RCT), for example, from the Netherlands where HPV DNA screening detected 70% more CIN3+ lesions than cytology screening (Bulkmans *et al*, 2007).

However, in recently published data from the ARTISTIC RCT undertaken in the United Kingdom (Kitchener *et al*, 2009a), the detection rate of CIN3+ lesions in the intervention arm (HPV testing combined with liquid-based cytology) was similar to that in the control arm (liquid-based cytology) (RR 0.97; 95% confidence interval (CI, 0.75–1.25). This observation was attributed to incomplete ascertainment of cases by observing that only 41.2% of HPV-positive/cytology-negative (HPV+/cyt−) women were ‘properly investigated’ (Sasieni *et al*, 2009). By correcting the observed CIN2+ detection for incomplete ascertainment, the sensitivity of HPV screening became higher than that for cytology, 94 *vs* 84%. Therefore, compliance with follow-up among women with positive screening tests needs to be carefully scrutinised to make an adequate comparison of the sensitivity of HPV testing measured in different trials.

In this paper, we present a thorough standardised overview regarding compliance with follow-up for all eight RCTs, with reported data from the baseline screening round. All of these RCTs aimed at measuring the sensitivity of HPV screening as compared with cytology screening, but women with positive screening tests were nevertheless recommended varying follow-up procedures. We therefore investigated the impact of the recommended follow-up procedure on the completeness of follow-up of screen-positive women, and subsequently assessed the effect of the completeness of follow-up on the measured sensitivity of HPV screening.

## Materials and methods

Eight RCTs comparing HPV DNA cervical screening with cytology screening had their baseline-round data published by February 2010. Six of these RCTs were undertaken in Europe (two in Italy, and one each in the Netherlands, United Kingdom, Sweden, and Finland), one in Canada, and one in India (Elfgren *et al*, 2005; Ronco *et al*, 2006a, 2006b, 2008, 2010; Bulkmans *et al*, 2007; Mayrand *et al*, 2007; Naucler *et al*, 2007, 2009; Kotaniemi-Talonen *et al*, 2008a, 2008b; Leinonen *et al*, 2009; Sankaranarayanan *et al*, 2009; Kitchener *et al*, 2009a, 2009b). From these publications, we extracted the data on the recommended follow-up procedure for screen-positive women, number of randomised women, number of women with positive screening tests, number of women with completed follow-up, and number of women with detected ⩾CIN3 at baseline. We used the following definitions of positive screening tests: ⩾ASCUS on cytology, ⩾1pg ml^−1^ HPV DNA on Hybrid Capture II HPV testing, and the cutoff point reported by the trialists using PCR GP5+/6+ HPV testing. In intervention arms in which both HPV and cytology were used as primary screening tests, women with at least one of the two tests being positive were considered screen-positive. We reported on the data from the Italian phase I RCT separately by age group, as in this RCT younger women (25–34 years) with abnormal screening tests had a recommended follow-up procedure different from that of older women (35–60 years).

For both arms of each RCT, we measured compliance with the recommended follow-up by calculating the proportion of screen-positive women who completed the recommended follow-up. For women referred for colposcopy we considered follow-up completed after the first colposcopy. For women not referred for colposcopy, we considered follow-up completed when all recommended repeated tests had been undertaken. Only follow-up with adequate repeated tests completed before the subsequent screening round was considered. We used the relative ⩾CIN3 detection as the indicator of relative sensitivity of HPV screening. The relative compliance and the 95% CI (Clayton and Hills, 1993), as well as the relative ⩾CIN3 detection and the 95% CI were calculated for the intervention group compared with the control group. A linear association between compliance and ⩾CIN3 detection was assessed using Pearson's correlation coefficient. To meet the assumptions for calculating this coefficient, the observed values for compliance and detection rate were transformed into logarithms.

## Results

### Impact of the recommended follow-up procedure on compliance with follow-up

The recommended follow-up procedures varied across RCTs, including a direct referral for colposcopy, or repeated testing at 6-, 12- or 18-month intervals with colposcopy recommended only in case of a positive outcome of the repeated testing (Tables 1 and 2). Proportion of women with completed follow-up could be at least partially determined from all RCTs, with the exception of the Finnish RCT (Tables 1 and 2).

Compliance with follow-up was dependent on the recommended follow-up procedure. Typically, around 90% of women complied with follow-up if they were immediately referred for colposcopy on the basis of their screening tests alone. In the intervention arms of the RCTs, this was found for women with ⩾ASCUS in Italy phase I, women with HPV+/cyt− tests in Italy phase I (35–60 years; Table 1), and HPV-positive women in Italy phase II, Canada, and India (Table 2). In the control arms of these RCTs, compliance of around 90% was observed for women with ⩾ASCUS in Canada and India, as well as in Italy phases I and II, in which the majority of women with ASCUS in the control arms (in seven out of nine centres) were directly referred for colposcopy.

Follow-up compliance was considerably lower when screen-positive women were recommended to first undergo repeated testing for 6 to 18 months after the initial screening. Among women with an HPV+/cyt− screening test (Table 1), 55% in the United Kingdom, 73% in Sweden, and 62% in Italy phase I (25–34 years) complied with repeated testing and a referral for colposcopy if recommended. In the intervention arm of the Dutch RCT, 58% of women with either HPV+/cyt− tests or ASCUS/LSIL complied with repeated testing. Data on compliance with repeated testing in the control arm were available from the Dutch RCT only, in which 66% of women with ASCUS/LSIL completed repeated testing.

The data on compliance with follow-up, as published so far, have been incomplete, and thus the relative compliance with follow-up could be calculated only from the data obtained from the Netherlands, Italy phases I and II, Canada, and India (Tables 1 and 2). Direct referral for colposcopy for screen-positive women in both arms of these RCTs (Italy phase I (35–60 years), Italy phase II, Canada, and India) produced non-significant differences in compliance with follow-up between the two arms. In the Netherlands, compliance with repeated testing was non-significantly lower in the intervention arm than in the control arm (0.88; 95% CI, 0.71–1.09). In Italy phase I (25–34 years), in which women with HPV+/cyt− tests were recommended repeated testing, whereas most women with ⩾ASCUS were directly referred for colposcopy, compliance in the intervention arm was significantly lower than in the control arm (0.84; 95% CI, 0.73–0.97). Therefore, with the exception of the latter RCT, the compliance with follow-up in the intervention arm was similar to the compliance in the control arm.

### The effect of completeness of follow-up on screening sensitivity

The eight RCTs showed surprisingly heterogeneous outcomes in terms of extra ⩾CIN3 detection with HPV testing. Data on relative ⩾CIN3 detection were available from all European RCTs (Tables 1 and 2). Two of these estimates were non-significantly below unity: UK 0.97 (95% CI, 0.75–1.25) and Italy phase II (25–34 years) 0.70 (95% CI, 0.37–1.34). Three estimates were statistically insignificantly increased: Sweden 1.31 (95% CI, 0.92–1.86), Italy phase I (35–60 years) 1.25 (95% CI, 0.78–2.01), and Finland 1.22 (95% CI, 0.78–1.92), whereas two estimates were statistically significantly increased: the Netherlands 1.70 (95% CI, 1.15–2.51) and Italy phase II 2.26 (95% CI, 1.42–3.58). The Canadian and Indian RCTs reported relative ⩾CIN2 detection only: 1.57 (95% CI, 0.76–3.24) and 0.87 (95% CI, 0.74–1.01), respectively.

Could this variation in relative ⩾CIN3 detection be explained by a variation in compliance with follow-up? As explained above, the complete compliance data were available from the Dutch, Italian, Canadian, and Indian RCTs only, whereas data on ⩾CIN3 detection were available from the European RCTs only. Relative compliance and relative ⩾CIN3 detection could therefore only be compared for the Dutch and Italian data (Tables 1 and 2). The correlation coefficient was 0.56 (*P*=0.44), suggesting a modest and non-significant correlation.

Given this paucity of complete data, we additionally considered only the data on the absolute level of compliance among women with HPV+/cyt− or HPV+ tests, which were available for all European RCTs except of the Finnish. Using these data, we calculated the correlation between compliance with follow-up for HPV+/cyt− (Sweden, United Kingdom, Italy phase I (25–34 years), and the Netherlands; Table 1) or HPV+ women (Italy phase II; Table 2), and the relative ⩾CIN3 detection. As a proxy for the compliance of HPV+/cyt− women in the Netherlands, we used the 58% compliance for combined ASCUS/LSIL and HPV+/cyt−. The correlation coefficient was 0.48 (*P*=0.33) in this case, that is, modest and non-significant correlation.

## Discussion

### Compliance with recommended follow-up

Although compliance with immediate referral for colposcopy was high (∼90%) in all RCTs with reported data, low compliance with repeated testing was common in all RCTs recommending this procedure. Among HPV+/cyt− women, 55% completed repeated testing in the UK RCT, 58% in the Dutch RCT, 62% in the Italian phase I RCT (25–34 years), and 73% in the Swedish RCT. Furthermore, in the control arm of the Dutch RCT, a comparable proportion, only 66%, of women completed the recommended repeated testing.

Our estimates of absolute compliance may have been affected by incomplete reporting of the RCT data. The United Kingdom, Italian, and Dutch RCTs did not systematically report the number of women with inadequate repeated tests. Because we assumed in such cases that all repeated tests were adequate, compliance with follow-up in terms of being ‘properly investigated’ may have been slightly overestimated. For the Dutch RCT, we could not determine, from the original report, whether the number with ‘completed repeat testing’ (Bulkmans *et al*, 2007) accounted for women undergoing colposcopy if recommended, while for Italy phase I RCT (25–34 years) it was not reported how many women with ASCUS with a recommendation for repeated testing did have repeated testing. In both cases, we assumed that all women completed the recommended follow-up tests.

For women testing HPV+/cyt− in the UK RCT, our estimate of compliance with follow-up, 54.6%, differed from the estimate reported earlier, 41.2% (Sasieni *et al*, 2009). The latter was calculated as 62.1% (the proportion of HPV+/cyt− women retested at 12 months) × 66.3% (proportion of women with persistent HPV on retest who had colposcopy). It did not include all women for whom the repeated test at 12 months was negative, and who were therefore ‘properly investigated’ (Sasieni *et al*, 2009) according to the protocol without undergoing colposcopy. Our estimate was additionally higher, although to a minor degree, because we accounted for women with a positive repeated HPV test at 12 months, who were given a choice to have a repeated HPV test at 24 months, and did not need to undergo colposcopy if they cleared the HPV infection by 24 months. This information was only provided in a later publication from the UK RCT (Kitchener *et al*, 2009b).

### Compliance with follow-up and sensitivity of HPV screening

In assessing the correlation between compliance and ⩾CIN3 detection, much emphasis has thus far been placed on the UK RCT. In this RCT, women with HPV+/cyt− screening tests were recommended repeated testing after 12 months, whereas women with ASCUS/LSIL were recommended repeated testing after 6 months. It was, however, not possible to independently determine whether this difference in the recommended time interval affected compliance and consequently the lack of extra detection of ⩾CIN3, as no follow-up data were reported for the control arm, nor have data for a similar group been reported in the routine screening statistics (The NHS Information Centre, Workforce and Facilities Team, 2009).

So far, virtually complete data on compliance have only been reported from the Dutch and Italian RCTs, with an estimated correlation of 0.56 (*P*=0.44) between compliance with follow-up and measured sensitivity of HPV screening. We used the data from the first reports on baseline data from the Italian RCTs (Ronco *et al*, 2006a, 2006b, 2008). The ⩾CIN3 detection was higher in a second report on baseline data (Ronco *et al*, 2010), but this report did not include updated data on compliance with follow-up.

Compliance was close to 90% in both arms of the Canadian and Indian RCTs. However, these RCTs reported only the relative ⩾CIN2 detection, being 1.57 (95% CI, 0.76–3.24) and 0.87 (95% CI, 0.74–1.01), respectively. Whereas the relative ⩾CIN3 detection could theoretically be above unity in the Canadian RCT, although not statistically significantly, this seems highly unlikely for the Indian RCT. The available data thus indicate a considerable variation in the relative ⩾CIN3 detection even in RCTs with good compliance with follow-up in both arms.

Owing to the lack of completely reported compliance data from all European RCTs, we instead investigated the correlation between the absolute level of completeness of follow-up among HPV+/cyt− or HPV+ women and the relative ⩾CIN3 detection. There was a considerable variation in this compliance—from 94% in Italy phase II to 55% in the United Kingdom—but this was not reflected in a corresponding variation in ⩾CIN3 detection, resulting in a low correlation coefficient of 0.48 (*P*=0.33).

However, in the interpretation of these outcomes, the paucity of the available data should be taken into account. To better elucidate the impact of compliance on the outcome of HPV screening, it is strongly recommended that comprehensive compliance data are published from all RCTs. Taken together, however, at present there is scant evidence from published reports to support the by now widely accepted view that the measured relative sensitivity of HPV screening is a simple reflection of relative compliance with follow-up.

### Appropriateness of adjustment for incomplete follow-up

If adjustment for incomplete follow-up is applied to RCT data, both arms should undergo adjustment so as to not bias the comparison. However, previously, only adjustment for women in the intervention arm who had an HPV+/cyt− test was applied (Sasieni *et al*, 2009). Furthermore, the CIN detection in colposcopically investigated women with repeated positive tests cannot be generalised to women with a positive screening test and no colposcopy. The latter group included women who did not need to undergo colposcopy, because their repeated tests were negative. The prevalence of CIN in these women may be substantially lower than in women with repeated positive HPV tests. Therefore, the outcomes of the reanalysis of the UK RCT data as carried out previously may have been biased.

Adjustment might, moreover, be problematic even when an unbiased estimate can be made. The European RCTs were undertaken to evaluate HPV screening in real-life settings. In this situation, an upwards correction of CIN detection due to incomplete compliance would be appropriate only if the level of compliance in the RCT was considerably lower than the level of compliance attainable in everyday screening practice. If, on the other hand, compliance with follow-up observed in the RCT is comparable with that attainable in the screening programme, CIN detection in the RCT can be seen as an acceptable estimate for the expected CIN detection. In this case, any further adjustment would lead to an artificial overestimation of ⩾CIN3 detection and would therefore affect the expected outcome of a shift from cytology to HPV screening.

## Conclusion

Incomplete compliance with recommended follow-up procedures for women with positive screening tests was found in several RCTs comparing HPV screening with cytology screening. Only some of the trials found the detection of ⩾CIN3 cases to be significantly increased in the HPV-screening arm. Follow-up compliance was dependent on the recommended follow-up procedures, but there was scant evidence of an unequivocal relationship between follow-up compliance and ⩾CIN3 detection. Adjustment of measured CIN detection on the basis of compliance data may not always be justifiable, and if adjustment is made, it should be used very judiciously.

## Figures and Tables

**Table 1:** Recommended follow-up, proportion of women with completed follow-up, and relative ⩾CIN3 detection in randomised controlled trials (RCTs) comparing HPV DNA screening with cytology screening: RCTs with HPV DNA and cytology testing in the intervention arm

	**United Kingdom**	**The Netherlands**	**Sweden**	**Italy phase 1**	**Italy phase 1**
Age (years)	20–64	29–56	32–38	25–34	35–60
					
*Control arm*					
⩾HSIL	Colp	Colp	Colp	Colp	Colp
LSIL	Repeat 6 and 12	Repeat 6 and 18	Repeat/colp	Colp	Colp
ASCUS	Repeat 6 and 12	Repeat 6 and 18	Repeat/colp	Colp/repeat 12	Colp/repeat 12
% Follow-up ⩾ASCUS	NA	ASCUS/LSIL: 66.3% (122/184)^a^	NA	93.1% (243/261)^b^	89.4% (531/594)^b^
					
*Intervention arm with HPV and cytology*					
⩾ASCUS	As control	As control	As control	Colp	Colp
HPV+/Cyt−	Repeat 12	Repeat 6 and 18	Repeat 12	Repeat 12	Colp
% Follow-up ⩾ASCUS	NA	NA	NA	94.3% (496/526)^c^	93.1% (832/894)^c^
% Follow-up HPV+/Cyt−	54.6% (915/1675)^d^	NA	73.0% (249/341)^e^	61.6% (314/510)^f^	93.5% (790/845)^c^
% Follow-up ⩾ASCUS or HPV+/Cyt−	NA	58.4% (268/459)^a^	NA	78.2% (810/1036)	93.3% (1622/1739)
					
*Intervention arm vs control arm*					
Relative % follow-up (95% CI)	NA	0.88 (0.71–1.09)^a^	NA	0.84 (0.73–0.97)	1.04 (0.95–1.15)
Relative detection rate of ⩾CIN3 (95% CI)	0.97 (0.75–1.25) ((233/18 386)/(80/6124))	1.70 (1.15–2.51) ((68/8575)/(40/8580))	1.31 (0.92–1.86) ((72/6257)/(55/6270))	0.70 (0.37–1.34) ((16/6002)/(22/5808))	1.25 (0.78–2.01) ((39/16 706)/(31/16 658))

Abbreviations: As control=women followed largely similar follow-up procedures as in the control arm; ASCUS=Atypical squamous cells of undetermined significance; CI=confidence interval; Colp=referral for colposcopy; Cyt=cytology; HPV=human papillomavirus; HSIL=High-grade squamous intraepithelial lesions; LSIL=Low-grade squamous intraepithelial lesions; NA=not available; Repeat=repeated testing, with the number indicating the period in months since screening.

a As reported by Bulkmans *et al* (2007), including in the intervention arm HPV+/cyt− women (*n*=280) and women with ASCUS/LSIL (*n*=179). Compliance was not reported for women with ⩾HSIL.

b Women who had colposcopy or women with ASCUS with a repeated test and colposcopy, if recommended, in two out of nine centres.

c Women who had colposcopy.

d Women with positive repeated HPV tests who had colposcopy (*n*=291) and women with a negative repeated HPV test (*n*=624).

e Women with positive repeated tests who had colposcopy (*n*=100) and women with negative repeated tests or a change in type of HPV (*n*=149).

f Women with adequate repeated cytology and HPV testing, who were either positive on one or both tests and had colposcopy (*n*=148) or had both tests negative (*n*=166). A total of 14 women had colposcopy immediately after screening; if these women are considered to have completed the follow-up, the proportion becomes 64.3% (328/510).

**Table 2:** Recommended follow-up, proportion of women with completed follow-up, and relative ⩾CIN3 detection in randomised controlled trials comparing HPV DNA screening with cytology screening: RCTs with stand-alone HPV testing in the intervention arm

	**Italy phase II**	**Finland**	**Canada**	**India**
Age (years)	25–60	30–60	30–69	30–59
				
*Control arm*				
⩾HSIL	Colp	Colp	Colp	Colp
LSIL	Colp	Colp	Colp	Colp
ASCUS	Colp/repeat 12	Repeat 12	Colp	Colp
% Follow-up ⩾ASCUS	87.6% (723/825)^a^	NA	91.4% (138/151)^b^	87.9% (1570/1787)^b^
				
*Intervention arm with stand-alone HPV*				
HPV+	Colp	LSIL+: Colp; other: repeat 12	Colp	Colp
% Follow-up HPV+	93.6% (1812/1936)^b^	NA	90.7% (284/313)^b^	89.1% (2505/2812)^b^
				
*Intervention arm vs control arm*				
Relative % follow-up (95% CI)	1.07 (0.98–1.16)	NA	0.99 (0.81–1.22)	1.01 (0.95–1.08)
Relative detection rate of ⩾CIN3 (95% CI)	2.26 (1.42–3.58) ((59/24 661)/(26/24 535))	1.22 (0.78–1.92) ((42/35 837)/(34/35 500))	1.57 (0.76–3.24) ((19/5095)/(12/5059))^c^	0.87 (0.74–1.01) ((318/27 192)/(345/25 549))^c^

Abbreviations: CI=confidence interval; Colp=referral for colposcopy; HPV=human papillomavirus; NA=not available; Repeat=repeated testing, with the number indicating the period in months since screening.

a Women who had colposcopy or women with ASCUS with a repeated test and colposcopy, if recommended, in two out of nine centres.

b Women who had colposcopy.

c Relative detection of ⩾CIN2; ⩾CIN3 not reported separately.
